# Time-based injection approach for monosegmented continuous flow systems and related techniques

**DOI:** 10.1155/S1463924696000247

**Published:** 1996

**Authors:** João Carlos de Andrade, Ronei Jesus Poppi, Aline Renée Coscione

**Affiliations:** Universidade Estadual de Campinas Instituto de Química C. P. 6154 Campinas SP 13083-970 Brazil


Journal of Automatic Chemistry, Vol. 18, No. 6 (November–December 1996) pp. 199–203

### Time-based injection approach for monosegmented continuous flow systems and related techniques

**João Carlos de Andrade, Ronei Jesus Poppi and Aline Renée Coscione**

*Universidade Estadual de Campinas, Instituto de Química, C. P. 6154, 13083-970, Campinas SP, Brazil*

*A time-based injection module for monosegmented continuous flow systems and related techniques, which uses three independently controlled solenoid valves, is described. A timer circuit employing three I.C. 555s and three TIP-121 transistors was constructed to control the injection module valves. The injection device was tested with non-reacting chemical systems (for example with a spectrophotometric standard and calcium flame emission) and with reacting conditions (for example the determination of Cr(VI), using diphenylcarbazide as colour reagent, and acid-base titration). The performance of this injection module demonstrates its suitability for everyday use.*

### Introduction

Most continuous flow analysis systems require the injection of a well-defined sample zone into the moving carrier stream. In contrast with the flow injection (FI) analysis technique (either usual FI [1, 2] or r-FI [3]), where the sample (or the reagent ) is injected to a continuous liquid carrier flow, the monosegmented continuous flow analysis (MCFA) system [4] was designed so that the sample is inserted into a carrier stream between air bubbles. Air-segmentation reduces the longitudinal dispersion of the sample along the flow path, reducing sample interaction with the carrier and permitting a longer sample residence time. As a consequence, this flow procedure is able to accommodate analytical methods involving relatively slow reactions without significant loss of sensitivity.

The approaches proposed for sample introduction into flow injection systems can be classified as volume-based or as time-based injection devices.

In the former case, the solution to be injected into the carrier stream is, at least for an instant, contained within an hermetically closed container, such as a valve bore or an external loop. The first prototype was a syringe with an hypodermic needle [5], later replaced by a syringe in combination with a flap valve [6]. More recently, sliding valve commutator [7] and microprocessed devices based on three-way or six-way valves [8, 9] have been employed as reliable alternatives.

Time-based injection devices are operated by pumping (or aspirating) the sample solution at a constant flow-rate into a well-defined section of a flow-through channel, for a fixed period of time, and inserting the sampled volume into a carrier stream by alternating the flow directions. This is usually done by using peristaltic pumps or rotating valves controlled by a timer [1, 2]. As the operation involves a combination of hydrostatic and hydrodynamic forces, this is known as 'hydrodynamic injection'.

Until now, only volume-based injection procedures using a sliding commutator or a six-way solenoid valve have been employed as sample injection devices for the MCFA systems. In this work, an alternative time-based injection device, which makes use of three three-way solenoid valves and a timer circuit based on three I.C. 555s, is presented.

### Experimental set-up

### *Prototype*

Figure 1 shows the timer circuit, the manifold and the arrangement for the solenoid valves used to build the time-based flow sample device. The basic idea is to connect three three-way valves so that the flow streams merge sequentially into a mixing point located at the normally closed (NC) end of the valve used for sample introduction. From this point, the flow stream is directed to the detector. The mixing plug (M in figure 1[*b*]) used for flow stream routing was made of Teflon, so that it had the lowest dead volume possible.

The principle of operation of such an injector device is quite simple. As shown in figure 1, by interrupting the carrier flow stream at valve 1 (V1), a gas (air) bubble (for MCFA) or reagent (for FI) is introduced into the manifold by a second valve (V2) which is sequentially turned on and off for periods of time t1, t2 and t3, according to the sequence shown in figure 1(*a*). During the time in which this second valve remains in the off position (normally open [NO] position), a third valve (V3) is turned on (brought to the NC position) for sample introduction. When valve V3 is turned off, valve V2 is again brought to its NC position, permitting the introduction of a second gas (air) bubble (for MCFA) or reagent (for FI). The injection cycle is finished after a period of time denoted $t$, where $t = t1 + t2 + t3$, when all valves are returned to their NO positions. As a consequence, valve V1 is kept activated (NC position) during the entire injection cycle.

This injection device was constructed using three three-way Cole Parmer electromechanical valves linked with 0·8 mm i.d. Teflon tubing through mixing point M. Each valve is brought into action for a fixed time by independently applying 12 V d.c. to it.

The timer circuit built to control the voltage to each valve is based on three independent I.C. 555s, operated

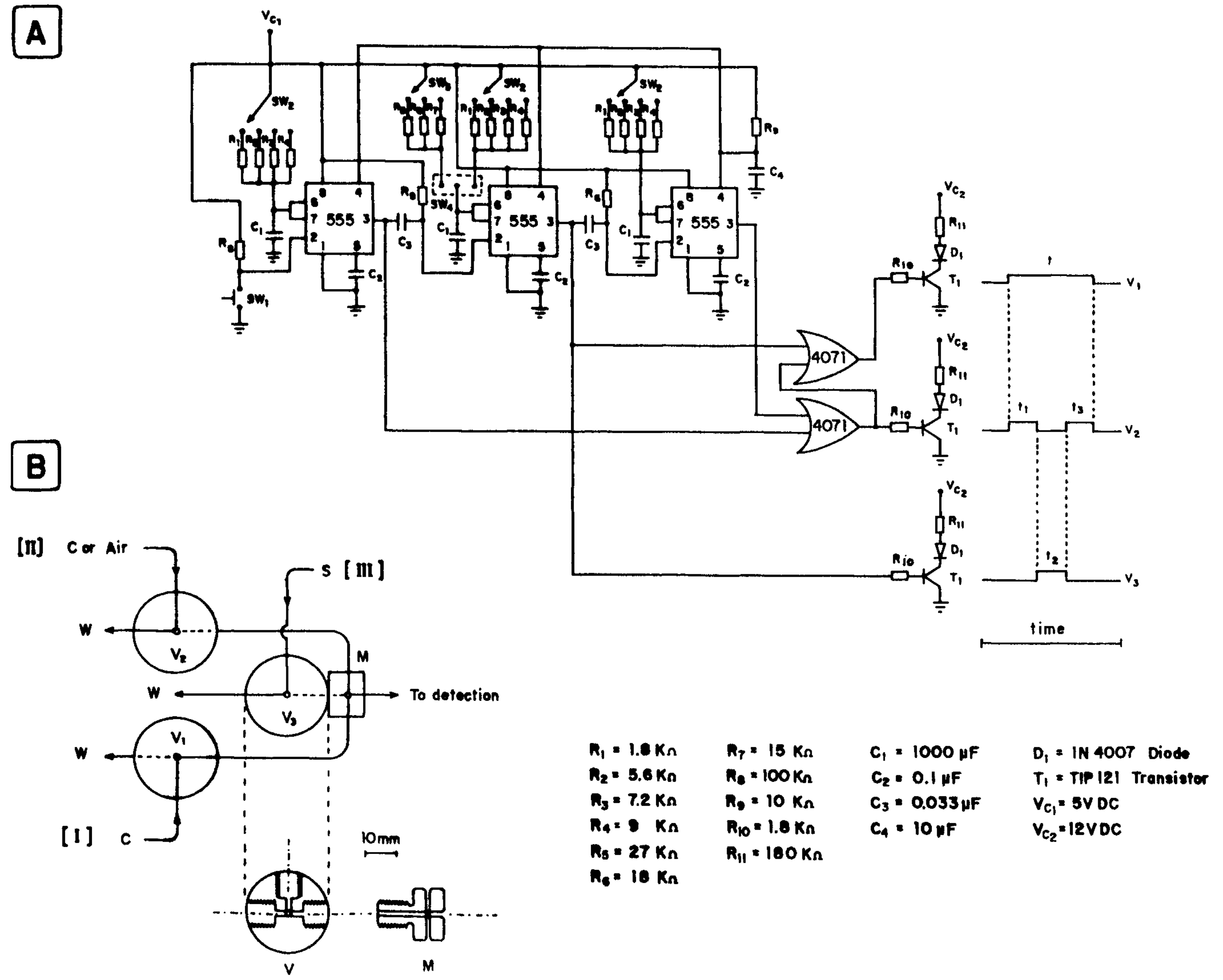

*Figure 1. The injection module. (a) Timer circuit. (b) Valve manifold.*

in conjunction with three TIP 121 transistors, as shown in figure 1 (*a*). The exit clock pulses from the I.C. 555s are directed to two NOR logic gates used to select the TTL level (low or high). When the TTL level is high, these pulses are allowed to reach the base of the transistors responsible for the valve activation.

According to this circuit, each I.C. 555 exit clock is controlled by a bank of resistors (R1 to R7) in figure 1[*a*]), activated from switches SW2 and SW3. These exit clocks control the activation time of each valve. Depending on the position of switch SW4, it is possible to select an extended time range for sample injection at V3. This occurs if switch SW4 is positioned in order to permit SW3 to be part of the circuit, exclusively changing the clock pulses from the I.C. 555, responsible for the activation time of V3.

It must be noted that this increment of time on V3 will automatically increase the time acting on V1, without any alteration to the operation time of V2. If the intention is to use approximately the same time of operation for all valves, SW4 must be positioned in such a way that SW3 becomes inoperative. Table 1 shows the time periods used in this protype.

This device can be used for monosegmented flow systems or for other continuous flow systems only by changing the external connections of the flow manifold.

### *Other apparatus, reagents and solutions*

Analytical-reagent grade chemicals and deionized water were used to prepare all solutions.

The standard stock solutions of $MnO_4^-$ ($1{\cdot}00 \times 10^{-2}$ mol $l^{-1}$), $Ca^{+2}$ (1000 μg $ml^{-1}$) and Cr(VI) (1000 μg $ml^{-1}$) were prepared by dissolving appropriate amounts of dried $KMnO_4$, $CaCO_3$ and $K_2Cr_2O_7$ in 1 l of water. The calcium carbonate was first dissolved in a minimum amount of HCl 1 + 1 (v/v) before dilution with water. The working standard solutions were obtained by dilution from stock.

The diphenylcarbazide (DPC) solution, used as the Cr(VI) colour reagent, was prepared by dissolving the compound in 20 ml of acetone and then diluting to

*Table 1. Valve operation times obtained with the resistor banks used in the prototype.*

| Activating switch | | Operation times (s) | | | Cycle times (s) |
|---|---|---|---|---|---|
| SW2 | SW3 | *t*1 | *t*2 | *t*3 | *t* |
| R1 | — | 2·16 | 2·21 | 2·21 | 6·58 |
| R2 | — | 6·56 | 6·93 | 7·06 | 20·55 |
| R3 | — | 8·70 | 9·14 | 9·27 | 27·16 |
| R4 | — | 10·81 | 11·49 | 11·72 | 34·02 |
| R1 | R5 | 2·20 | 33·48 | 2·21 | 37·89 |
| R2 | R5 | 6·60 | 33·55 | 7·11 | 47·26 |
| R3 | R5 | 8·72 | 33·62 | 9·36 | 51·70 |
| R4 | R5 | 10·82 | 33·59 | 11·69 | 56·10 |
| R1 | R6 | 2·21 | 22·38 | 2·19 | 26·78 |
| R2 | R6 | 6·55 | 22·38 | 7·04 | 35·97 |
| R3 | R6 | 8·66 | 22·51 | 9·34 | 40·51 |
| R4 | R6 | 10·78 | 22·60 | 11·70 | 45·08 |
| R1 | R7 | 2·20 | 18·90 | 2·18 | 23·28 |
| R2 | R7 | 6·61 | 18·89 | 7·17 | 32·67 |
| R3 | R7 | 8·72 | 18·78 | 9·33 | 36·83 |
| R4 | R6 | 10·79 | 18·91 | 11·73 | 41·43 |

*t*1 and *t*3 are the injection times for V2; *t*2 is the injection time for V3; *t* is the total injection cycle time, in which V1 remains activated. The values presented are the average of 10 independent measurements. R1, R2, R3, R4, R5, R6 and R7 are the resistors described in figure 1(*a*).

500 ml with water [10]. Prior to its reaction with Cr(VI), this solution is mixed on-line with 0·8 mol $l^{-1}$ $H_2SO_4$. The procedures followed for both MCFA and FI determinations are those described in the literature [4, 10].

The flow titrations [1, 11] were performed by injecting a small volume of HCl solution, with concentrations ranging from $10^{-2}$ to $10^{-3}$ mol $l^{-1}$, into a constant flow stream of 0·998 mol $l^{-1}$ NaOH standard solution containing a few drops of a 0·001% (m/v) bromotymol blue solution. This flow stream is directed through a 730 µl reaction cell and then to the flow-cell for signal detection.

The fluids were pumped at a flow rate of 1·7 ml $min^{-1}$ using an eight-roller Rainin Rabbit peristaltic pump and Tygon pump tubing. Polyethylene ($v = 600$ µl) or glass ($v = 2500$ µl) tubing was employed as mixing or reaction coils in the flow manifold, according to the experimental needs. The signals were measured by a Zeiss PM2A spectrophotometer and recorded at the appropriate maximum wavelength, using an 80 µl Zeiss flow cell with an optical pathlength of 10 mm. In the flow flame emission experiments with $Ca^{2+}$, the flame photometer (Micronal) was operated as directed by the manufacturer.

### Results and discussion

Screening experiments using $KMnO_4$ solutions with concentrations ranging from $5{\cdot}00 \times 10^{-5}$ mol $l^{-1}$ to $2{\cdot}50 \times 10^{-4}$ mol $l^{-1}$ were done to evaluate the overall performance of the injection device under usual conditions for both MCFA and FI.

Either the monosegmented system or the flow injection system can be easily implemented by simply changing the gas (air) to a reagent solution or a carrier at valve V2. No other changes in the valve arrangement or the timer circuit are required.

The results of 10 consecutive injections of a $1{\cdot}00 \times 10^{-2}$ mol $l^{-1}$ $KMnO_4$ solution, using a reaction coil of 2500 µl and *t*1 : *t*2 : *t*3 settings at 6·61s : 18·89s : 7·17s, respectively, operated as monosegmented flow, indicated a maximum relative standard deviation of 1% for peak height measurements. The response curves were obtained by averaging quadruplicate consecutive absorbance transients peaks over the concentration range tested and resulted in linear relationships for both MCFA ($A = 0{\cdot}005 + 0{\cdot}188C_{MnO_4^-}$ [mol $l^{-1}$]; $r = 0{\cdot}9996$) and for FI ($A = -0{\cdot}007 + 0{\cdot}092C_{MnO_4^-}$ [mol $l^{-1}$]; $r = 0{\cdot}9997$). Both curves were obtained under the same experimental conditions, using the MCFA manifold, since a permeation cell is essential for bubble removal under MCFA operation. Thus, the FI technique showed to be less sensitive, as a result of its inherent on-line sample dilution.

This injection device presents an inherent dead volume at about 9 µl, due to the longitudinal hole at the Teflon mixing plug attached to valve V3, which is responsible for the flow connection with the reaction manifold. This dead volume needs to be as small as possible to avoid response variations on the transient flow peak heights. These variations are less sensitive for FI measurements due to bolus on-line dilution, becoming important only if consecutive samples with large differences in concentration are introduced in sequence. Although these deviations are not relevant for most cases, corrections can be easily made if needed, considering that only the first signal may be affected and that the flow techniques usually make use of at least triplicate injections. Also the use of smaller solenoid valves and drilling smaller longitudinal holes in the plug M will minimize the possible effects of the dead volume.

As the injection is time-based, the major problems are expected to be related to the propelling system, because changes in the flow rates during the injection period may cause variations in the signals. However, no problems of this type were observed using good quality peristaltic pumps, such as that used here.

The injection device was also tested under other flow system situations, such as the MCFA and FI determinations of Ca(II) by flame emission photometry and the spectrophotometric determination of Cr(VI) with diphenylcarbazide [4, 10], as well as the flow injection titration of HCl solutions with standard NaOH [1, 11]. The results are summarized in table 2.

Other flow applications for this time-based injection system such as FI double or triple zone injection [12] and MCFA single bubble injection [13] can be derived and are shown in figure 2. These can be implemented by changing only the reagents (or air) and the carrier input lines of each valve. FI operation using two valves can be implemented by disabling V2.

*Table 2. Results of reacting and non-reacting chemical systems used to test the performance of the time-based injection device under FI and MCFA operating conditions.*

| Analytical procedure | | Experimental set-up | Calibration curve | Concentration tested |
|---|---|---|---|---|
| Spectrophotometric FI titration | HCl with standard NaOH | 2 solenoid valve<br>$\lambda = 620$ nm<br>$t1 : t2 : t3 = 2{\cdot}16 : 2{\cdot}21 : 2{\cdot}21$(s) | $\Delta t = 7{\cdot}928 - 2{\cdot}638\ (-\log C_{HCl})$<br>$r = 0{\cdot}9999$ | $10^{-3} < C_{HCl} < 10^{-2}$ (mol $l^{-1}$) |
| MCFA flame photometry | $Ca^{+2}$ | 3 solenoid valves<br>glass coil = 2500 µl<br>$t1 : t2 : t3 = 10{\cdot}79 : 18{\cdot}91 : 11{\cdot}73$(s) | $I = 0{\cdot}525 + 0{\cdot}244C_{Ca^{2+}}$<br>$r = 0{\cdot}9995$ | $10 < C_{Ca^{2+}} < 80$ (µg $ml^{-1}$) |
| FI flame photometry | $Ca^{+2}$ | 2 solenoid valves<br>glass coil = 2500 µl<br>$t1 : t2 : t3 = 10{\cdot}79 : 18{\cdot}91 : 11{\cdot}73$(s) | $I = 0{\cdot}255 + 0{\cdot}136C_{Ca^{2+}}$<br>$r = 0{\cdot}9992$ | $10 < C_{Ca^{2+}} < 100$ (µg $ml^{-1}$) |
| MCFA spectro-photometric determination Cr(VI) | Cr(VI)/ DPC | 3 solenoid valves<br>$\lambda = 540$ nm<br>$t1 : t2 : t3 = 6{\cdot}61 : 18{\cdot}89 : 7{\cdot}17$(s) | $A = 0{\cdot}005 + 0{\cdot}597C_{Cr(VI)}$<br>$r = 0{\cdot}9993$ | $0{\cdot}200 < C_{Cr(VI)} < 2{\cdot}00$ (µg $ml^{-1}$) |
| FI spectro-photometric determination Cr(VI) | Cr(VI)/ DPC | 2 or3 solenoid valves<br>$\lambda = 540$ nm<br>$t1 : t2 : t3 = 6{\cdot}61 : 18{\cdot}89 : 7{\cdot}17$(s) | $A = 0{\cdot}001 + 0{\cdot}198C_{Cr(VI)}$<br>$r = 0{\cdot}9983$ | $0{\cdot}200 < C_{Cr(VI)} < 2{\cdot}00$ (µg $ml^{-1}$) |

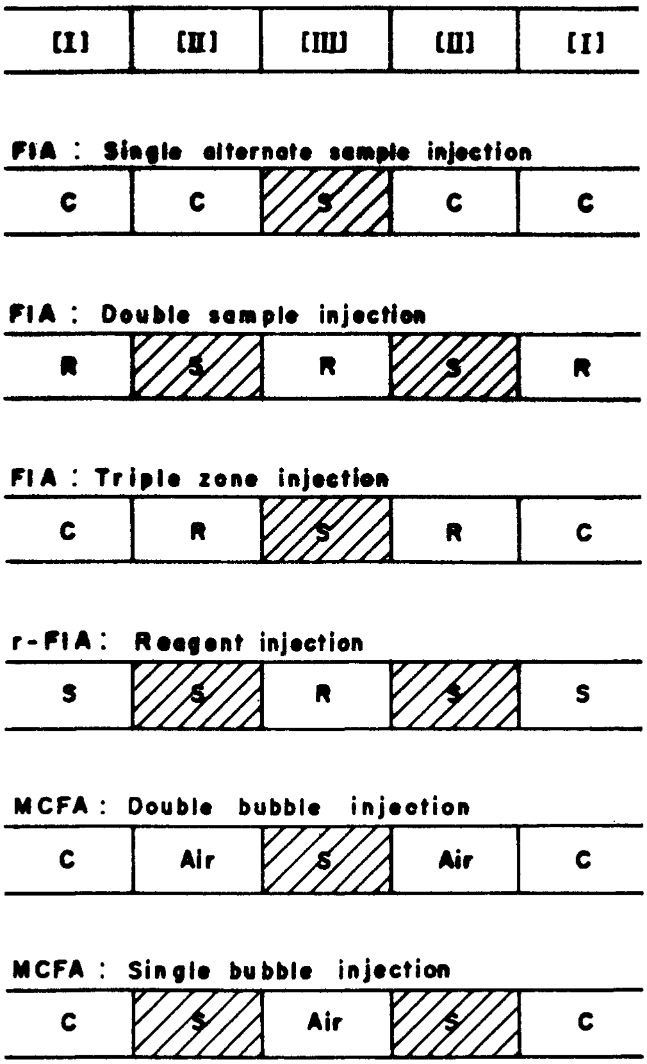

*Figure 2. Other injection procedures which can be implemented without changes in the injection module. C = carrier stream; S = sample; R = reagent;* [*I*], [*II*] and [*III*] *= valve input lines, as shown in figure 1(b).*

Another important feature for the proposed injection device is that its operation does not depend on a dedicated computer for its control, making possible its use in situations where a computer is not available for full time use. On the other hand, the use of a computer to control the interface TTL output levels will make the proposed injection system much more versatile, since it could assume any value of time for the valve operations. This could permit an alternative means of control of the valves, giving rise to possible new injection configurations and applications.

### Conclusion

This paper shows that it is possible to construct an automatic time-based injection module for monosegmented continuous flow system and related techniques, using only three three-way solenoid valves and a simple timer circuit. This is a low cost module whose unique operational set-up was tested for MCFA and FI using both non-reacting and reacting chemical systems, confirming its utility in practical situations, without changes in the electronic circuit or valve arrangements.

### References

1. Ruzicka, J. and Hansen, E. H., *Flow Injection Analysis*, 2nd edn (Wiley, New York, 1988).

2. Cases, V. M., and de Castro, M. D. L., *Analisis por Inyeccion en Flujo* (Universidad de Cordoba, Cordoba, 1984).

3. Johnson, K. S. and Petty, R. L., *Analytical Chemistry*, **54** (1982), 1185.

4. Pasquini, C. and de Oliveira, W., *Analytical Chemistry*, **57** (1985), 2575.

5. Ruzicka, J. and Hansen, E. H., *Analytica Chimica Acta*, **78** (1975), 145.

6. Stewart, J. W. B., Ruzicka, J., Bergamin Fo., H. and Zagatto, E. A. G., *Analytica Chimica Acta*, **82** (1976), 371.

7. Bergamin Fo, H., Medeiros, J. X., Reis, B. F. and Zagatto, E. A. G., *Analytica Chimica Acta*, **101** (1978), 9.

8. Toli, J., *Analyst*, **112** (1987), 106.

9. Farias, L. C. and Pasquini, C., *Journal of Automatic Chemistry*, **13** (1991), 143.

10. de Andrade, J. C., Rocha, J. C., Pasquini, C. and Baccan, N., *Analyst*, **108** (1983), 621.

11. Hansen, E. H. and Ruzicka, J., *Journal of Chemical Education*, **56** (1979), 677.

12. Zagatto, E. A. G., Bahia Fo, O., Guiné, M. F. and Bergamin, Fo, H., *Analytica Chimica Acta*, **181** (1986), 265.

13. Reis, B. F., Arruda, M. A. Z., Zagatto, E. A. G. and Ferreira, J. R., *Analytica Chimica Acta*, **206** (1988), 253.

